# Effectiveness of *Mentha piperita* in the Treatment of Infantile Colic: A Crossover Study

**DOI:** 10.1155/2012/981352

**Published:** 2012-07-12

**Authors:** João Guilherme Bezerra Alves, Rita de Cássia Coelho Moraes de Brito, Telma Samila Cavalcanti

**Affiliations:** Instituto de Medicina Integral Prof. Fernando Figueira (IMIP), Rua dos Coelhos, 301 Boa Vista, 52050-080 Recife, PE, Brazil

## Abstract

*Background*. Infantile colic is a distressing and common condition for which there is no proven standard treatment. *Objective*. To compare the efficacy of *Mentha piperita* with simethicone in treatment for infantile colic. *Methods*. A double-blind crossover study was performed with 30 infants attending IMIP, Recife, Brazil. They were randomized to use *Mentha piperita* or simethicone in the treatment of infantile colic during 7 days with each drug. Primary outcomes were mother_s opinion about responses to the treatment, number of daily episodes of colic, and time spent crying, measured by a chronometer. Mann-Whitney and chi-square tests were used to compare the results. This study was previously approved by the Ethical Committee in Research at IMIP. *Results*. At baseline daily episodes of infantile colic was 3.9 (±1.1) and the mean crying time per day was 192 minutes (±51.6). At the end of the study daily episodes of colic fell to 1.6 (±0.6) and the crying duration decreased to 111 (±28) minutes. All mothers reported decrease of frequency and duration of the episodes of infantile colic and there were no differences between responses to *Mentha piperita* and simethicone. *Conclusions*. These findings suggest that *Mentha piperita* may be used to help control infantile colic. However, these results must be repeated by others studies.

## 1. Introduction

 Infantile colic is a common condition in the first months of life reaching around 5% to 28% of infants [1]. Usually it appears after the second week of life and disappears around the fourth month of life [2]. It is defined as a crisis of paroxysmal attacks of irritability, restlessness, or crying more than three hours a day, three days a week for more than three weeks in an otherwise well-fed, healthy baby [3].

Etiopathogenesis and better treatment for infantile colic remain unknown [4–6]. Gastrointestinal, psychosocial, and neurodevelopmental disorders have been suggested as the causes of infantile colic [7]. Gastrointestinal disorder has been implicated in colic because of the infant's grimacing and leg position during a crying spell [2, 8]. Hyperperistalsis, aerophagia, and allergy to cow's milk have been speculated as gastrointestinal causes [7, 8]. Therefore, various pharmacologic agents, herbal products, and chiropractic manipulation have been experimented for this determination [4, 5, 9].

Simethicone is widely used for colic in infants in many countries. This drug is a mixture of polydimethylsiloxanes that reduce the surface tension of air bubbles. Simethicone is relatively safe because it is not absorbed into the bloodstream. It reduces abdominal discomfort by promoting the clearance of excessive gas along the gastrointestinal tract [10].

*Mentha piperita *is an herbal well known and has been used for a variety of symptoms and diseases [11]. *Mentha piperita* has a long history of safe use both in medicinal preparations and as a flavoring agent in foods [12]. In folk medicine, it has been used as an antiemetic, antiparasitic, anti-inflammatory, antibacterial, and antispasmodic including treatment for infantile colic [13–15]. *Mentha piperita* has a spasmolytic effect on the smooth muscle of the gastrointestinal tract [16]. However, no studies had focused on *Mentha piperita* efficacy and safety in treatment for infantile colic. In a double-blind crossover study, we compare the efficacy of *Mentha piperita* with simethicone in treatment for infantile colic.

## 2. Material and Methods 

### 2.1. Subjects and Eligibility Criteria

Infants aged 15 to 60 days, exclusively breastfeeding and treated at Instituto de Medicina Integral Professor Fernando Figueira (IMIP), Recife, Brazil, between March 2011 and December 2011, were eligible for the study entry. Infantile colic was characterized according to Wessel et al. [3] criteria: paroxysmal attacks of irritability, restlessness, or crying for at least three hours a day, and occurring more than three days a week for a period of three weeks. Complete physical examination was performed on all infants routinely to exclude other possible crying reasons. Exclusion criteria were illiterate mothers, living outside the metropolitan region of Recife, prematurity or low birth weight (<2,500 g), failure to thrive, gastrointestinal disorders, and currently infectious, allergic or metabolic disease. Children were also excluded if they were receiving some type of treatment (drug, herbal products or chiropractic manipulation).

Primary outcomes were evaluated by mother's opinion about responses to the treatment, number of daily episodes of colic, and time spent crying, measured by a chronometer. Secondary outcomes were number of milk regurgitation, vomiting, diarrhea, constipation, and drowsiness.

All children's parents signed the consent form. This study was approved by the Ethical Committee in Research at IMIP (CEP/IMIP, number/09).

### 2.2. Study Intervention

This study was carried out using a crossover double-blind design. Each child underwent an intervention for 14 days. The infants were firstly randomized in two groups to receive formulation of leaves of the *Mentha piperita* (liquid drops; 1 drop per Kg body weight) or simethicone (liquid drops; 2.5 mg per Kg body weight) daily for a period of 7 days. After the first 7 days of study and a period of wash out for 3 days, all the children had their medication alternated and were followed for more 7 days. Repeated visits were scheduled for the seventh and seventeenth days after the first visit. On the seventh day visit, the medication was returned to the hospital and another pair of medication were distributed. When patients did not return to the hospital on the seventh day, a home visit was conducted by a researcher. During wash out period, the parents were oriented to use paracetamol for colic treatment. During visits, the infant was clinically examined.

*Mentha piperita* and simethicone were identical in weight, smell, color, taste, and package. The drugs were arranged in numbered pairs, and were randomly designated by letters A or B. All researchers and parents were unaware of the treatment administered. The allocation sequence and randomization list were computer-generated using the “Randomized” program (http://www.randomized.com/). Parents filled out a daily structured form with notes on the number and time of crying episodes, administration of medications, and unintended effects. A chronometer was previously provided to the parents to determine the crying time of their children.

In order to determine the sample size, a preliminary trial was performed with 15 children to evaluate the average and variance of the difference in infantile colic frequency between *Mentha piperita* and simethicone. The sample size calculation was based on the finding of 50 minutes difference between groups in reduction of crying time, which was considered a clinically relevant difference. Considering a significance level set at 0.05 and a power of 80% and an estimated standard deviation in groups of 50 minutes, 22 patients would be necessary. Presuming a loss of 30% of the sample, the number was increased to 30 participants.

### 2.3. Statistical Analysis

The Mann-Whitney and chi-square tests were used to compare continuous variables and categorical variables, respectively. The proportions of patients with effective and ineffective responses in each group were compared using the chi-square test. In all comparisons it was provided with a *P* value <0.05 as statistically significant. Data were expressed as means and ranges. All the analysis was performed by SPSS 12 software (SPSS Inc, Chicago, IL).

## 3. Results

Among 313 infants studied, 30 (7.7%) were diagnosed with infantile colic. Three of them were excluded based on the exclusion criteria, and the remaining were randomized. After the beginning of the trial, no parental withdrawal occurred. There was no adverse effect due to the interventions in the present study protocol.

30 infants aged 8 to 56 days (33 ± 11.1) were studied. The average weight and height were 4.650 g (±415) and 54.2 cm (±3.0), respectively. The maternal age ranged from 14 to 32 years (22.7 ± 5.4) and they had 10.4 years (±2.5) of schooling. All mothers had received prenatal care and 16 (53.3%) had undergone cesarean section.

At baseline daily episodes of infantile colic were 3.9 (±1.1) and the mean of crying time per day was 192 minutes (±51.6). At the end of the study (17 days) daily episodes of colic fell to 1.6 (±0.6) and the crying duration decreased to 111 (±28) minutes. All mothers reported decrease of frequency and duration of the episodes of infantile colic and there were no differences between responses to *Mentha piperita* and simethicone (Table 1).

## 4. Discussion

The prevalence of infantile colic in this study (7.7%) was similar to other studies [1, 7, 8]. The responses to both treatments, *Mentha piperita* and simethicone, did not show statistical differences according to parents evaluation. All parents reported lower frequency and duration of infantile colic in their children during the period of the study. Unintended effects were not observed in this present study. Although there is still some controversy, clinical trials have shown that simethicone, an over-the-counter drug, is safe and helps to control infantile colic [17–19]. We did not find studies with the same objectives to ours to compare results. The antispasmodic activity of *Mentha piperita* has been tested mainly in animals and the spasmolytic effect of peppermint oil on the intestinal musculature appears to involve calcium antagonism [20, 21]. A study with peppermint oil capsule seemed to reduce the pain that children experienced during the acute phases of irritable bowel syndrome [22–24]. Savino et al. in a randomized trial showed that colic in breastfed infant improves with an extract based on *Matricaria recutita*, *Foeniculum vulgare,* and *Melissa officinalis* [25].

To our best knowledge, this is the first time that a clinical trial study evaluated the efficacy of *Mentha piperita* on the infantile colic treatment. For diseases on some aspects of physiopathology are unknown as infantile colic, the use of this medication on the same patient offers some advantages. In this study, we used a double-blind crossover design. This design in this study offers the control of several variables that may influence the presence of colic as genetic factors, environmental, emotional, and eating maternal habits. We also include only exclusively breastfeeding infants for better controlling of food variable. We believe that all this contributed to strengthening our findings.

Our study has some limitations. At first, the natural history of infantile colic is to improve with time. Although we have used a crossover design, the possible effect of time on the improvement of infantile colic could not be totally eliminated. Secondly, we could not measure objectively the compliance of the drugs. All parents filled out a structured form but we cannot guarantee the accuracy of this information.

In conclusion, our findings suggest that *Mentha piperita* may be used to help control infantile colic. However, these results must be proven by other studies.

## Figures and Tables

**Table 1:** Effects of *Mentha piperita* and simethicone on infantile colic.

Variables	*Mentha piperita*		Simethicone		*P*-value
	*n*	% (SD)	*n*	% (SD)
Mother evaluation					
Slightly improved	12	40.0	12	40.0	0.271^∗^
Greatly improved	6	20.0	5	16.7	
Completely improved	12	40.0	13	43.3	

Daily colic episodes	1.7	(±0.5)	1.5	(±0.6)	0.456^∗^
Daily colic duration	114	(±26)	109	(±29)	0.787^∗∗^
Duration of the colic after medication	17.0	(13.8)	16.0	(10.0)	0.860^∗∗^

^∗^Fisher's Exact Test ^∗∗^Mann-Whitney Test.
